# Supplementary material for: A Delphi technique toward the development of a cognitive intervention framework module for breast cancer survivors with cognitive impairment following chemotherapy
Source: PLoS One. 2022 Nov 17;17(11):e0277056. doi: 10.1371/journal.pone.0277056 (PMC9671464; doi:10.1371/journal.pone.0277056)
Supplement: S1 Table — (PDF) [file pone.0277056.s001.pdf]

S1 Table. Results from Delphi Round 1 (N=35).

Items	M (SD)	% Rated 2-3
Objectives of attention training		
1. Are the objectives stated clear and sensible?	2.23 (0.60)	91
2. Are the objectives practical to accomplish?	2.26 (0.44)	100
3. Do Malaysian breast cancer survivors have the insight to improve their attention functioning?	1.94 (0.42)	94
Definition of attention		
1. Are the definitions provided precise and easy to understand?	2.06 (0.68)	80
2. Is the language used suitable for Malaysian breast cancer survivors?	1.97 (0.75)	77
Activities for attention		
1. Are the instructions provided easy to understand?	2.03 (0.71)	77
2. Are the activities suggested appropriate?	2.09 (0.51)	91
3. Do the activities reflect the objectives of attention?	2.14 (0.49)	94
4. Are Malaysian therapists capable of delivering these activities?	2.23 (0.55)	94
5. Can Malaysian breast cancer survivors complete these activities?	1.97 (0.51)	86
6. Do the activities not cause compliance problems or cause breast cancer survivors to drop out?	1.94 (0.42)	89
Approaches to the rehabilitation of attention: N-Back task		
1. Is the rationale provided clear?	2.03 (0.57)	86
2. Are the procedures suggested easy to understand?	1.63 (0.65)	60
3. Do the tasks reflect the objectives of attention training?	2.06 (0.48)	91
4. Are Malaysian therapists capable of delivering the tasks?	2.03 (0.57)	86
5. Can Malaysian breast cancer survivors complete the tasks?	1.89 (0.47)	83
6. Do the activities not cause compliance problems or cause breast cancer survivors to drop out?	1.80 (0.47)	97
Approaches to the rehabilitation of attention: Time Pressure Management (TPM)		
1. Is the rationale provided clear?	2.17 (0.57)	91
2. Are the procedures suggested easy to understand?	2.03 (0.57)	86
3. Do the strategies reflect the objectives of attention training?	2.23 (0.55)	94
4. Are Malaysian therapists capable of delivering the strategies?	2.23 (0.60)	91
5. Can Malaysian breast cancer survivors complete the strategies?	2.03 (0.51)	89
6. Do the strategies not cause compliance problems or breast cancer survivors to drop out?	1.91 (0.45)	92

S1 Table. (continued).

Items	M (SD)	% Rated 2-3
Objectives of memory training		
1. Are the objectives stated clear and sensible?	2.29 (0.52)	97
2. Are the objectives practical to accomplish?	2.31 (0.47)	100
3. Do Malaysian breast cancer survivors have the insight to improve their memory functioning?	1.91 (0.51)	83
Definition of memory		
1. Are the definitions provided precise and easy to understand?	2.20 (0.63)	89
2. Is the language used suitable for Malaysian breast cancer survivors?	2.03 (0.45)	91
Approaches to the rehabilitation of memory: External compensation- Memory notebook		
1. Is the basis of the memory notebook provided clear?	2.31 (0.53)	97
2. Are the sections suggested in the memory notebook easy to understand?	2.23 (0.55)	94
3. Are the examples provided for each stage of memory notebook procedures easy to understand?	2.20 (0.53)	94
4. Does the use of a memory notebook reflect the objectives of memory training?	2.31 (0.47)	100
5. Are Malaysian therapists capable of delivering the activities?	2.34 (0.54)	97
6. Can Malaysian breast cancer survivors complete the activities?	2.20 (0.58)	92
7. Do the strategies not cause compliance problems or breast cancer survivors to drop out?	1.91 (0.51)	89
8. Are the techniques practical to be applied to breast cancer survivors' daily life?	2.00 (0.59)	83
Approaches to the rehabilitation of memory: Memory Strategy Training- Association techniques		
1. Is the basis of the techniques provided clear?	2.29 (0.46)	100
2. Are the techniques suggested easy to understand?	2.29 (0.52)	97
3. Are the examples provided for each technique easy to understand?	2.20 (0.53)	94
4. Does the use of the techniques reflect the objectives of memory training?	2.20 (0.47)	97
5. Are Malaysian therapists capable of delivering the techniques?	2.29 (0.57)	94
6. Can Malaysian breast cancer survivors complete the activities?	1.97 (0.51)	86
7. Do the strategies not cause compliance problems or breast cancer survivors to drop out?	1.83 (0.57)	86
8. Are the techniques practical to be applied to breast cancer survivors' daily life?	2.06 (0.54)	89

S1 Table. (continued).

Items	M (SD)	% Rated 2-3
Approaches to the rehabilitation of memory: Memory Strategy Training- Organizational and elaboration techniques		
1. Is the basis of the techniques provided clear?	2.26 (0.56)	94
2. Are the techniques suggested easy to understand?	2.17 (0.66)	86
3. Are the examples provided for each technique easy to understand?	2.14 (0.49)	94
4. Does the use of the techniques reflect the objectives of memory training?	2.23 (0.55)	94
5. Are Malaysian therapists capable of delivering the techniques?	2.34 (0.54)	97
6. Do the strategies not cause compliance problems or breast cancer survivors to drop out?	1.89 (0.32)	89
7. Are the techniques practical to be applied to breast cancer survivors' daily life?	2.09 (0.51)	91
Approaches to the rehabilitation of memory: Activities for memory		
1. Are the instructions provided easy to understand?	2.09 (0.51)	97
2. Are the activities suggested appropriate?	2.23 (0.49)	100
3. Do the activities reflect the objectives of the training?	2.26 (0.44)	100
4. Are Malaysian therapists capable of delivering the techniques?	2.26 (0.51)	97
5. Can Malaysian breast cancer survivors complete the activities?	2.09 (0.37)	97
6. Do the strategies not cause compliance problems or breast cancer survivors to drop out?	1.89 (0.40)	86

Notes: 3= Strongly agree, 2= Agree.
