# Supplementary material for: A Delphi technique toward the development of a cognitive intervention framework module for breast cancer survivors with cognitive impairment following chemotherapy
Source: PLoS One. 2022 Nov 17;17(11):e0277056. doi: 10.1371/journal.pone.0277056 (PMC9671464; doi:10.1371/journal.pone.0277056)
Supplement: S2 Table — (PDF) [file pone.0277056.s002.pdf]

S2 Table. Major themes from coding the responses obtained in Round 1 (N=35).

Component	Mentioned by
1. Other approaches/strategies/ techniques to improve memory	
The cognitive intervention module is comprehensive	31%
Integration of electronic devices	9%
Errorless learning	8%
Cognitive remedial therapy	6%
Mindfulness training	3%
Cogmed Working Memory Training	3%
2. Other approaches/strategies/ techniques to improve attention	
The cognitive intervention module is comprehensive	14%
Mindfulness training	11%
Cognitive remedial therapy	6%
Psychopharmacology	6%
Functional activities	6%
3. Appropriateness of the cognitive intervention module to be incorporated into clinical practice	
Very likely	46%
Moderately	43%
4. Experts responsible for administering the cognitive intervention module	
Occupational therapist and clinical psychologist	43%
Occupational therapist only	34%
Clinical psychologist only	23%
5. The appropriate time to administer the cognitive intervention module	
Post-chemotherapy	57%
3 to 6 months following chemotherapy	6%
One year following chemotherapy	3%
6. Monitoring effectiveness of the cognitive intervention module	
Baseline, during, and post-intervention	31%
Baseline and post-intervention	26%
During follow-up cognitive training	23%
Continuous monitoring	20%
7. Logistic issues that will impact the implementation of the cognitive intervention module	
Transportation	40%
Time constraint	20%
Financial issues	9%
Proper setting to administer the intervention	9%
Accessibility to treatment	6%
Family support	6%
Cost of therapy	3%

S2 Table. (continued).

Component	Mentioned by
8. Potential benefits, risks, and burdens to the therapist in implementing the cognitive intervention module	
The cognitive intervention module provides a standard guideline	60%
Time constraint	31%
A therapist needs appropriate training in administering the cognitive intervention module	17%
Expand the scope of clinical services	11%
Extra workload	6%
9. Potential benefits, risks, and burdens to the breast cancer survivor if the cognitive intervention module is to be administered	
Improve cognitive functioning	49%
Improve the quality of life	26%
Time constraint	14%
Improve daily function	11%
Lack of motivation and support	11%
Improve psychological well-being	6%
Improve work performance	6%
10. Ethical issues arise if the cognitive intervention module to be administered	
None	34%
Compulsory to obtain consent from a breast cancer survivor	9%
