# Supplementary material for: A Delphi technique toward the development of a cognitive intervention framework module for breast cancer survivors with cognitive impairment following chemotherapy
Source: PLoS One. 2022 Nov 17;17(11):e0277056. doi: 10.1371/journal.pone.0277056 (PMC9671464; doi:10.1371/journal.pone.0277056)
Supplement: S3 Table — (PDF) [file pone.0277056.s003.pdf]

S3 Table. Items retained from the Round 1 questionnaire.

Items
Objectives of attention training
1. Are the objectives stated clearer?
2. Are the objectives more practical to accomplish?
Definition of attention
1. Are the definitions provided precise and easy to understand?
Activities for attention
1. Are the instructions provided easy to understand?
Approaches to the rehabilitation of attention: N-Back task
1. Are the procedures suggested easy to understand?
Approaches to the rehabilitation of attention: Time Pressure Management (TPM)
1. Are the procedures suggested easy to understand?
Objectives of memory training
1. Are the objectives stated clearer?
2. Are the objectives more practical to accomplish?
