# Supplementary material for: A Delphi technique toward the development of a cognitive intervention framework module for breast cancer survivors with cognitive impairment following chemotherapy
Source: PLoS One. 2022 Nov 17;17(11):e0277056. doi: 10.1371/journal.pone.0277056 (PMC9671464; doi:10.1371/journal.pone.0277056)
Supplement: Attachment — Submitted filename: reviewer_comments_PONE-D-22-07018.docx [file pone.0277056.s004.docx]

Title: Title is development of module, but the finding form this work only the survey/feedback from the experts. I don’t agree with the title. I strongly agree that this work is “a preliminary work” towards the intervention module. If it is a development of module, the module should be tested or subjected to trials among the CRCI patient.

Abstract: Discussion and interpretation of data is limited. No proper intro and statement of problem. Finding and conclusion just highlight about the successful of Delphi technique with limited number of participant and expert (35), somehow not proven in text the effectiveness of this technique like comparison to other approach/ analysis.

Introduction, Line 67 – 144:

1) The literature work of related field is limited. The readers would like to know the existence of other work in implementation of CRCI intervention as well as under ACRM and other intervention framework under international commission and societies. Yes, you did mention EF, INSIGHT, PCHP, MAAT, ACTIVE ect., but can you give their findings and stats? This will help reader interpret your problem statements which is why you need to conduct the CRCI intervention using Delphi technique.
2) The author main tool/approach is Delphi technique is not well-discussed
3) In introduction, please provide background of CRCI any stats and figure, if you can give table by states by cases. It will benefit the reader.

Material and methods, Line 120: Does this design referring Figure 1? In this section, what is the approach or criteria in selecting the experts. The number 33 of experts passing round 3 could be yield a bias result and need basis or justification in terms of experts selection.

Result and discussion: Limited discussion. This part should discuss the development of the intervention module. But author only interpret the figures of survey data from the expect. If not just revise the title accordingly to the contents.
