# Supplementary material for: A Delphi technique toward the development of a cognitive intervention framework module for breast cancer survivors with cognitive impairment following chemotherapy
Source: PLoS One. 2022 Nov 17;17(11):e0277056. doi: 10.1371/journal.pone.0277056 (PMC9671464; doi:10.1371/journal.pone.0277056)
Supplement: Attachment — Submitted filename: 10 July EDITORs comments.docx [file pone.0277056.s006.docx]

**(PONE-D-22-07018-R1)**

| **No.** | **Comment(s)** | **Action(s)** | **Editor Comments-R1** |
| --- | --- | --- | --- |
| **1.** | Title:  Title is development of module, but the finding form this work only the survey/feedback from the experts. I don’t agree with the title. I strongly agree that this work is “a preliminary work” towards the intervention module. If it is a development of module, the module should be tested or subjected to trials among the CRCI patient. | Thank you very much for your suggestion.  *Accepted and made changes.*  All authors agree with your suggestion. The new title is on the first page of the manuscript and it is highlighted in **PINK.** | A Delphi technique to consensus building of a cognitive intervention module for breast cancer  survivors with cognitive impairment following chemotherapy.  The title above still reflecting on the author’s work one a developed module.  As commented previously, it should never be a module development. A survey technique (Delphi) to consensus building is relevant only for a preliminary work to develop an intervention framework or to validate after the module have been developed.  My title suggestion:  A Delphi technique toward a development of a cognitive intervention framework module for breast cancer  survivors with cognitive impairment following chemotherapy. |
| **2.** | Abstract:  Discussion and interpretation of data is limited. No proper intro and statement of problem. Finding and conclusion just highlight about the successful of Delphi technique with limited number of participant and expert (35), somehow not proven in text the effectiveness of this technique like comparison to other approach/ analysis apparently employed.  As such, concerns about inappropriate normative references remain, and continue to threaten the meaningfulness and validity of your findings. | Thank you very much for your suggestion.  *Accepted and made changes.*  All authors agree with your suggestion. Kindly go to **Abstract**, then **objectives** and changes are highlighted in **GREEN**.  Also, all authors agree with your expert opinion and therefore, we critically evaluate the advantages and disadvantages of the Delphi method and made comparison with other group methods such as a focus group. Kindy go to the **Critical reflection on the study and the study results** and changes are highlighted in **YELLOW**. | Results and conclusion have not been highlighted in abstract. None has been addressed. It is important to highlight the main finding from this study. Otherwise it will be an ambiguous work.  Eg. What criteria are all agreed by experts?  Experts agreed on attention and  memory training objectives?  On suitability of the language and clarity of the instructions?  Critical reflection on the study and the study results: Acceptable |
| **3.** | Introduction, Line 67-144: 1) The literature work of related field is limited. The readers would like to know the existence of other work in implementation of CRCI intervention as well as under ACRM and other intervention framework under international commission and societies. Yes, you did mention EF, INSIGHT, PCHP, MAAT, ACTIVE ect., but can you give their findings and stats? This will help reader interpret your problem statements which is why you need to conduct the CRCI intervention using Delphi technique.  2) The author main tool/approach is Delphi technique is not well-discussed.  3) In introduction, please provide background of CRCI any stats and figure, if you can give table by states by cases. It will benefit the reader. | Thank you very much for your suggestion.  *Accepted and made changes.*  All authors agree with your suggestion.  Previously published systematic review of cognitive intervention that evaluated the effects of cognitive rehabilitation programs on cognitive impairment among the various population of participants with non-central nervous system (CNS) cancer patients have produced inconsistent findings (Fernandes, Richard, & Edelstein, 2019; Treanor et al., 2016).  For example, Treanor and colleagues (2016) did not make equivocal recommendations on the effectiveness of non-pharmacological interventions in ameliorating cognitive function following chemotherapy treatment for mixed cancer patients. This systematic review used the cognitive intervention recommended by the Cognitive Rehabilitation Task Force of the Brain Injury Interdisciplinary Special Interest Group (BI-SIG) of the American Congress of Rehabilitation Medicine (ACRM) similar to our present study but among **mixed cancer patients**.  In 2021 (Syed Alwi et al., 2021), we published a systematic review that used the cognitive intervention recommended by the Cognitive Rehabilitation Task Force of the Brain Injury Interdisciplinary Special Interest Group (BI-SIG) of the American Congress of Rehabilitation Medicine (ACRM) among **breast cancer survivors**.  At present, there are only 10 studies conducted investigated the effectiveness of cognitive intervention for breast cancer survivors. The published studies reported the effect size (Cohen’s *d*). Therefore, all authors agree to use the effect size in reporting the effectiveness of the cognitive intervention.  Kindly go to the **Introduction** and changes are highlighted in **YELLOW**.  *Accepted and made changes.*  All authors agree with your suggestion.  Kindly go to the **Introduction** and changes are highlighted in **PINK**.  *Accepted and made changes.*  All authors agree with your suggestion. We understand that the table will benefit the reader. However, all authors made the decision not to include the table because it is not our primary objective of our present study. Therefore, we provide the ranges for both prevalence and incidence of cognitive impairment.  To the best of our knowledge there are a lot of published studies that are available examining the prevalence and incidence of cognitive impairment with the earliest study conducted in 1998.  Many of newer studies in this field has been cited in the **Introduction**.  Kindly go to the **Introduction** and changes are highlighted in **GREEN**. We have added the prevalence and incidence of cognitive impairment. | Please include the comments given here in the manuscript text body  Then copy and paste put it in your introduction, so reader know the background of the study. Indicate the details of results and citation of your previous work.  Please address the details of those 10 studies in summary or table. It would be preferable in Table.  Line 79-83. Please extend the discussion by giving few examples of cognitive impairment.  Acceptable  Acceptable |
| **4.** | Material and methods, Line 120:  Does this design referring Figure 1? In this section, what is the approach or criteria in selecting the experts. The number 33 of experts passing round 3 could yield a bias result and need basis or justification in terms of experts selection. | Thank you very much for your suggestion.  *Accepted and made changes.*  All authors agree with your suggestion. Kindly go to the **Material and methods** and **Study designs** and changes are highlighted in **PINK**.  Kindly go to the **Material and methods** and **Participants** and approach or criteria in selecting the experts are highlighted in **TURQUOISE**.  In principle, the validity of the results obtained from the Delphi method does not root in the statistical significance but rather in  the iterative process of expert opinion development and consensus building (Cole et al., 2013).  Therefore, we highlighted the issue of statistical significance in the **Introduction** (highlighted in **PINK**) and critically evaluated the advantages and disadvantages of the Delphi method and made a comparison with other group methods such as a focus group. Kindy go to the **Critical reflection on the study, and the study results** and changes are highlighted in **YELLOW**. | Acceptable  Acceptable |
| **5.** | Result and discussion: Limited discussion. This part should discuss the development of the intervention module. But author only interpret the figures of survey data from the expect. If not just revise the title accordingly to the contents. | *Accepted and made changes.*  All authors agree with your suggestion. We revised the title of our manuscript. |  |
|  |  |  | Line 264-280: Redundant. Not significant to be discussed in this part. These have been mentioned in introduction/problem statement |
|  |  |  | Disussion - Sufficient |
|  |  |  | Conclusion –  Line 383-384 -: Justification on this multiracial/mutiecthnic. Can it be applied to other southeast asia region?  Line 384: This module? This work is preliminary work not accredited or etsbalished module. Please change “the module” with “the proposed framework” or “the module elements”. Please revise throughout the study from this comment.  In this section, please provide one MAIN conclusion.  Practicable?  Sensible/relevant? |
