# Supplementary material for: A Delphi technique toward the development of a cognitive intervention framework module for breast cancer survivors with cognitive impairment following chemotherapy
Source: PLoS One. 2022 Nov 17;17(11):e0277056. doi: 10.1371/journal.pone.0277056 (PMC9671464; doi:10.1371/journal.pone.0277056)
Supplement: Attachment — Submitted filename: EDITOR DR MOHAMAD SYAZWAN.docx [file pone.0277056.s008.docx]

**Editor (PONE-D-22-07018-R1)**

**A Delphi technique toward the development of a cognitive intervention framework module for breast cancer survivors with cognitive impairment following chemotherapy**

| **No.** | **Editor Comments-R1** | **Action(s)** |
| --- | --- | --- |
| **1.** | The document entitled "EDITOR.docx" are given details of author response/actions toward the reviewer comment, however the document entitled "MAIN TEXT FILE WITH TRACK CHANGES" only contains some of the corrected text. It is difficult to follow some of the author response eg; (All authors agree with your suggestion. Kindly go to the Introduction and changes are highlighted in PINK), but nothing is highlighted in PINK colour. So please check it ASAP. | Dear Editor,  We would like to apologise for the inconvenience that we have caused.  Also, to accelerate the process, we added **PAGE NUMBER** under **ACTION(S)**. |
| **2.** | A Delphi technique to consensus building of a cognitive intervention module for breast cancer  survivors with cognitive impairment following chemotherapy.  The title above still reflecting on the author’s work one a developed module.  As commented previously, it should never be a module development. A survey technique (Delphi) to consensus building is relevant only for a preliminary work to develop an intervention framework or to validate after the module have been developed.  My title suggestion:  A Delphi technique toward a development of a cognitive intervention framework module for breast cancer  survivors with cognitive impairment following chemotherapy. | Thank you very much for your suggestion.  *Accepted and made changes.*  All authors agree with your suggestion.  The new title is on **PAGE 1** of the manuscript and it is highlighted in **PINK.** |
| **3.** | Results and conclusion have not been highlighted in abstract. None has been addressed. It is important to highlight the main finding from this study. Otherwise it will be an ambiguous work.  Eg. What criteria are all agreed by experts?  Experts agreed on attention and memory training objectives?  On suitability of the language and clarity of the instructions?  Critical reflection on the study and the study results: Acceptable | Thank you very much for your suggestion.  *Accepted and made changes.*  All authors agree with your suggestion. Kindly go to **PAGE 2**, **Abstract**, then **Results** highlighted in **YELLOW** and **Conclusion** highlighted in **TURQUOISE**.  Thank you very much for the expert opinions given earlier to improve the manuscript. |
| **4.** | Please include the comments given here in the manuscript text body  Then copy and paste put it in your introduction, so reader know the background of the study. Indicate the details of results and citation of your previous work.  Please address the details of those 10 studies in summary or table. It would be preferable in Table.  Line 79-83. Please extend the discussion by giving few examples of cognitive impairment. | Thank you very much for your suggestion.  *Accepted and made changes.*  All authors agree with your suggestion.  Kindly go to **PAGE 4** and **PAGE 5,** then the **Introduction** and changes are highlighted in **TURQUOISE**.  *Accepted but unable to make changes.*  We would like to apologize for not being able to include the table requested because all authors believed that it can be considered self-plagiarism. Therefore, we provided the citation of our systematic review and summarized the studies in the current text.  Thank you very much for your suggestion.  *Accepted and made changes.*  All authors agree with your suggestion. Kindly go to **PAGE 4**, the **Introduction** and changes are highlighted in **YELLOW**. |
| **5.** | Line 264-280: Redundant. Not significant to be discussed in this part. These have been mentioned in introduction/problem statement. | Thank you very much for your suggestion.  *Accepted and made changes.*  All authors agree with your suggestion. Kindly go to **PAGE 10**, the **Discussion**. The points in lines 264 to 280 were removed. |
| **6.** | Discussion - Sufficient | Thank you very much for the expert opinions given earlier to improve the **Discussion**. |
| **7.** | Conclusion –  Line 383-384 -: Justification on this multiracial/mutiecthnic. Can it be applied to other southeast asia region?  Line 384: This module? This work is preliminary work not accredited or etsbalished module. Please change “the module” with “the proposed framework” or “the module elements”. Please revise throughout the study from this comment.  In this section, please provide one MAIN conclusion.  Practicable?  Sensible/relevant? | Thank you very much for your suggestion.  *Accepted and made changes.*  All authors agree with your suggestion. Kindly go to **PAGE 13**, to the **Conclusion** and changes are highlighted in **TURQUOISE**.  Thank you very much for your suggestion.  *Accepted and made changes.*  All authors agree with your suggestion. We have revised our manuscript accordingly. Changes can be seen throughout the text with **“framework module”** or **“proposed framework”** highlighted in **GREEN**.  Thank you very much for your suggestion.  *Accepted and made changes.*  All authors agree with your suggestion. Kindly go to **PAGE 13** the **Conclusion** and changes are highlighted in **YELLOW**. |
