# Supplementary material for: A Delphi technique toward the development of a cognitive intervention framework module for breast cancer survivors with cognitive impairment following chemotherapy
Source: PLoS One. 2022 Nov 17;17(11):e0277056. doi: 10.1371/journal.pone.0277056 (PMC9671464; doi:10.1371/journal.pone.0277056)
Supplement: Attachment — Submitted filename: EDITOR DR MOHAMAD SYAZWAN.docx [file pone.0277056.s009.docx]

**Editor (PONE-D-22-07018-R3)**

**A Delphi technique toward the development of a cognitive intervention framework module for breast cancer survivors with cognitive impairment following chemotherapy**

| **No.** | **Editor Comments-R3** | **Action(s)** |
| --- | --- | --- |
| **1.** | **Abstract, Line 61-65** - Please revise the conclusion in the abstract. The current conclusion in abstract should be in ONE LINE, which addressing the final conclusion of your work. In my humble evaluation, the first line (61-63) just explaining what your work is aiming at, objective etc. and the second line is recommendations. My suggestion/example: This study found out that a cognitive intervention framework module for breast cancer survivors with cognitive impairment following chemotherapy can be successfully developed/ or feasible to be implemented using Delphi technique/study/approach. | Dear Editor,  We would like to express our gratitude for reviewing our manuscript.  Thank you very much for your suggestion.  *Accepted and made changes.*  All authors agree with your suggestion.  Kindly go to **PAGE 2 (LINE 61-63), Abstract**, then **Conclusions** and changes are highlighted in **GREEN**. |
